# Preconception Weight Change and Pregnancy Outcomes After Bariatric Surgery

**DOI:** 10.3390/jcm15155851

**Published:** 2026-07-27

**Authors:** Taylor Guthrie, Sandra Lee, Alka Kothari, William Pinzon Perez, Sailesh Kumar, Helen Truby, Susan de Jersey

**Affiliations:** 1 Faculty of Health, Medicine & Behavioural Sciences, University of Queensland, Herston, QLD 4029, Australia; alka.kothari@uq.edu.au (A.K.); sailesh.kumar@mater.uq.edu.au (S.K.); h.truby@uq.edu.au (H.T.); susan.dejersey@health.qld.gov.au (S.d.J.); 2 Dietetics & Foodservices, Royal Brisbane Women’s Hospital, Herston, QLD 4029, Australia; 3 Maternity Services, Caboolture Hospital, Caboolture, QLD 4510, Australia; sandra.lee@health.qld.gov.au; 4 Obstetrics & Gynecology, Redcliffe Hospital, Redcliffe, QLD 4020, Australia; 5 Queensland Cyber Infrastructure Foundation, Fortitude Valley, QLD 4006, Australia; w.pinzonperez@qcif.edu.au; 6 Mater Research Institute, University of Queensland, Brisbane, QLD 4072, Australia

**Keywords:** bariatric surgery, weight loss, pregnancy, obesity, birth weight, premature birth

## Abstract

**Background/Objectives:** Consensus guidelines recommend delaying conception for at least 12 months following bariatric surgery to achieve maternal weight stability; however, the impact this has on perinatal outcomes is poorly understood. The aim of this study was to explore the impact of bariatric surgery-to-conception interval and maternal preconception weight change on birth weight and gestational age at birth. **Methods:** Women aged 18–45 years with singleton pregnancies after bariatric surgery were recruited to a prospective, observational study before 23 weeks’ gestation. Participants self-reported the surgery-to-conception interval and weight change in the six months before conception. Preconception weight change was calculated as a percentage of difference compared to weight at conception. Linear regression assessed predictors of birth weight and gestational age. **Results:** Data from 68 participants (mean age 32 ± 4.8 years) were analyzed. The median birth weight was 3368 (514) g with 7% (5/68) SGA infants. Women with SGA infants had greater preconception weight loss (12% vs. 0%, *p* = 0.029), while surgery-to-conception intervals were similar (*p* = 0.255). Preconception weight change and gestational age were independent predictors of birth weight (*p* < 0.001), accounting for 58% of its variance. Surgery-to-conception interval did not predict birth weight (*p* = 0.986). The median gestational age at birth was 39 (2.1) weeks, with 7% (5/68) preterm births. Neither preconception weight change nor surgery-to-conception interval predicted gestational age at birth. **Conclusions:** Preconception weight change appears to be more influential than surgery-to-conception interval for birth weight and birth of an SGA infant. This suggests a need for individualized counseling regarding timing of pregnancy after bariatric surgery.

## 1. Introduction

Maternal bariatric surgery improves fertility and reduces the risk of gestational diabetes mellitus (GDM), macrosomia, gestational hypertension, and pre-eclampsia [1,2,3]. However, it increases rates of small-for-gestational-age (SGA) infants, preterm birth, and maternal micronutrient deficiencies, [1,2,3] which can induce epigenetic changes which may affect long-term health for both mother and child [4].

The timing of pregnancy after bariatric surgery is a key consideration [5]. Modern procedures like sleeve gastrectomy, single-anastomosis gastric bypass, and Roux-en-Y gastric bypass induce significant weight loss (20–35% of body weight) by reducing intake and impairing nutrient absorption, and can cause vomiting or postprandial syndrome, particularly in the first post-operative year [6,7]. Given the increased nutritional demands of pregnancy, conceiving within 12 months of bariatric surgery is thought to heighten the risk of adverse outcomes arising from compromised nutritional intake and micronutrient status [8,9]. International guidelines recommend that women should delay pregnancy for 12–24 months after bariatric surgery to maximize weight loss and enable weight stabilization before conception [10,11]. However, the evidence underpinning this recommendation comes from only one study [12]. Other publications examining outcomes related to surgery-to-conception interval have not reported maternal weight status (weight stable, gaining or losing weight) at conception [13,14,15,16]. Qualitative research has reported consumer frustration with current recommendations to delay pregnancy, particularly among women seeking bariatric surgery to address subfertility [17]. Delaying pregnancy must be balanced against the decline in fertility associated with increasing maternal age and the likelihood of regaining weight which occurs in almost 40% of women post-operatively [18,19]. Therefore, the aim of this study was to explore the impact of both surgery-to-conception interval and preconception weight change on birth weight and gestational age at birth.

## 2. Materials and Methods

This study was part of a larger prospective cohort study examining maternal nutrition after bariatric surgery across four maternity hospitals in South East Queensland, Australia (June 2022–February 2024) [20,21]. In addition to the present study, this cohort study investigated maternal dietary intake [20], micronutrient status [22], and gestational weight gain [21]. Ethical approval was granted by the Metro North Health Human Research and Ethics Committee (EC00168).

Pregnant women aged 18–45 years who were <23 weeks’ gestation with prior bariatric surgery were eligible to participate. Women with multiple pregnancy or pre-existing co-morbidities that influenced nutrition or birth outcomes (including Type 1 and 2 Diabetes Mellitus and kidney disease) and those who were unable to participate in English were excluded.

Eligible women were referred by health professionals or contacted via text message with a link to study information. The first author provided study details and obtained informed verbal consent to participate over the telephone. Participant information and consent forms were completed electronically via REDCap. In accordance with practice guidelines, each participant had access to specialist care during their pregnancy, including referral to a dietitian, monitoring of maternal micronutrient status each trimester, and serial ultrasound monitoring for fetal growth [20,23]. Participants entered the draw to win one of five AU$20 grocery vouchers as thanks for their participation.

Participants reported the following data via a REDCap survey at study enrolment: weight (six months prior to conception and pre-surgery), height, age, type and date of bariatric surgery and other demographic information. Gestational weight gain was self-reported at 36 weeks’ gestation. Medical records were used to confirm surgical procedure and date, and weight at conception and at 36 weeks. Records of preconception weight were not available in medical records. To minimize recall bias, implausible values were verified with individual participants, and all participants were encouraged to confirm their preconception weight using records from their general practitioner or other healthcare providers where available. Smoking, use of any assisted conception techniques, previous preterm birth (<37 weeks’ gestation) and pregnancy outcomes (offspring sex, birth weight, and gestational age, mode of birth, complications) were extracted from medical records.

The surgery-to-conception interval was estimated by subtracting 280 days from the expected date of confinement and categorized as <12 months or ≥12 months. Body Mass Index (BMI) was calculated as weight divided by height squared (kg/m^2^). To account for variation in BMI between participants, weight change was expressed as a percentage of conception weight at three and six months preconception using the following equation:

$$\left(\frac{\mathrm{Conception}\mathrm{weight}-\mathrm{weight}\mathrm{three}\mathrm{or}\mathrm{six}\mathrm{months}\mathrm{preconception}}{\mathrm{Conception}\mathrm{weight}}\right)\times 100$$

This weight change was expressed as either a negative value (indicating weight loss) or a positive value (indicating weight gain leading up to conception), and was considered stable if it remained within ±5% of conception weight. This definition of weight stability was developed considering normal weight fluctuations among adult women in the absence of a broadly accepted definition of weight stability post-bariatric surgery [24,25,26]. Gestational weight gain was calculated by subtracting conception weight from weight at 36 weeks’ gestation and compared to the Institute of Medicine’s guidelines based on BMI at conception [27]. SGA (<10th percentile) and large-for-gestational age (LGA, >90th percentile) offspring were identified using Fenton [28] growth charts considering gestational age at birth and offspring sex.

Data were analyzed using SPSS (v.30, 2024). Continuous variables were tested for normality (Shapiro–Wilk test). Normally distributed variables were reported as mean ± standard deviation (SD) and compared using independent samples *t*-tests and ANOVA; non-normally distributed data were summarized as median with interquartile range (IQR) and compared using Mann–Whitney U and Kruskal–Wallis H tests. Spearman’s correlation assessed associations between preconception weight change, surgery-to-conception interval, birth weight, and gestational age. Linear regression (forward stepwise selection, entry *p* ≤ 0.05, removal *p* ≥ 0.10, up to 20 iterations) examined predictors of birth weight and gestational age, adjusting for parity, smoking, gestational hypertension, GDM and gestational age at birth (birth weight only). A *p*-value of <0.05 was considered statistically significant.

## 3. Results

**Figure 1:**
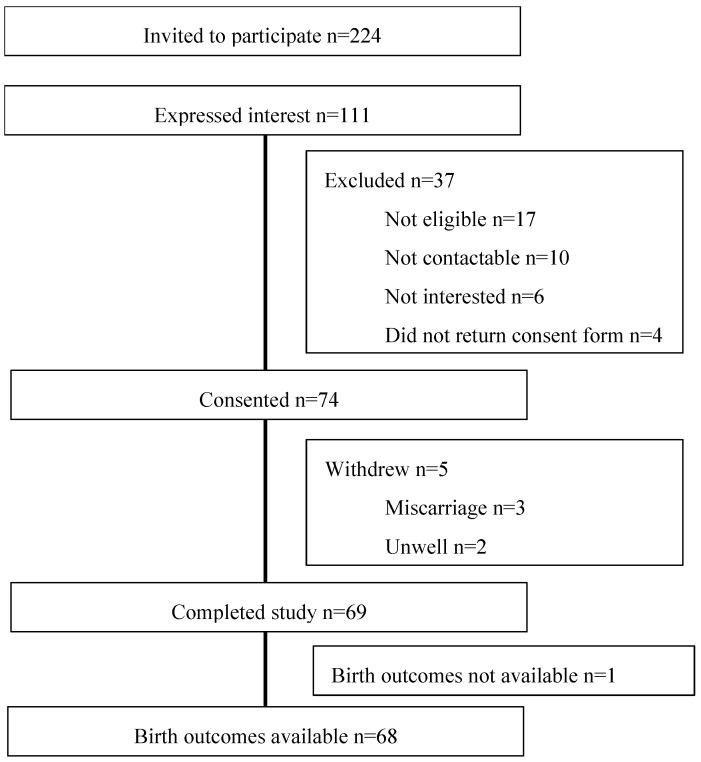
Participant flowchart.

The study included 68 participants (Figure 1), of whom 68% (46/68) had undergone a sleeve gastrectomy with a median surgery-to-conception interval of 31 (50) months (Table 1). Preconception weight change varied; however, women who conceived less than 12 months after surgery had a greater weight change at three (−10.1 (6)% vs. 0.0 (4)%, *p* < 0.001) and six months preconception (−19.1 (15)% vs. 0.0 (4)%, *p* < 0.001) and less gestational weight gain compared to women who conceived after 12 months (−0.2 ± 3.7 kg vs. 8.4 ± 7.1 kg, *p* = 0.006; Table 2).

### 3.1. Offspring Birth Weight

Maternal and neonatal health outcomes are summarized in Table 3. Most participants gave birth vaginally; however, 42% (28/67) underwent a cesarean section. Cesarian sections were primarily elective (43%, 12/28), and other indications are detailed in Supplementary Table S1. The median birth weight was 3368 (514) g, which had a moderate, positive correlation with weight change at six months preconception (R^2^ = 0.30, *p* = 0.016) but not at three months preconception (R^2^ = 0.17, *p* = 0.200). Median birth weight was lower among women with inadequate gestational weight gain (3197 (413) g), compared with those with recommended (3525 (297) g) and excess weight gain (3510 (564) g, *p* = 0.024). Birth weight was similar between mothers who had undergone restrictive bariatric surgery (sleeve gastrectomy and gastric band) and bypass procedures (*p* = 0.328).

Stepwise linear regression identified predictors of birth weight. In the first step, gestational age at birth was statistically significant at *F*(1, 57) = 66.37, *p* < 0.001. In the second step, weight change six months preconception was entered into the model, which was also statistically significant at *F*(2, 56) = 40.86, *p* < 0.001. The final model, including gestational age at birth and weight change six months preconception, accounted for a substantial proportion of the variation in birth weight (R^2^ = 0.593, adjusted R^2^ = 0.579). Gestational age at birth (*t =* 8.95, *p* < 0.001, β = 0.773) and preconception weight change (*t =* 2.76, *p* = 0.008, β = 0.239) had positive standardized coefficients, suggesting greater gestational age and weight gain preconception were both associated with higher birth weight. Surgery-to-conception interval (*t =* −0.458, *p* = 0.649), GDM (*t =* 0.127, *p* = 0.899), smoking (*t =* −0.160, *p* = 0.874) and gestational hypertension (*t =* 0.450, *p* = 0.654) were excluded from the model.

**Figure 2:**
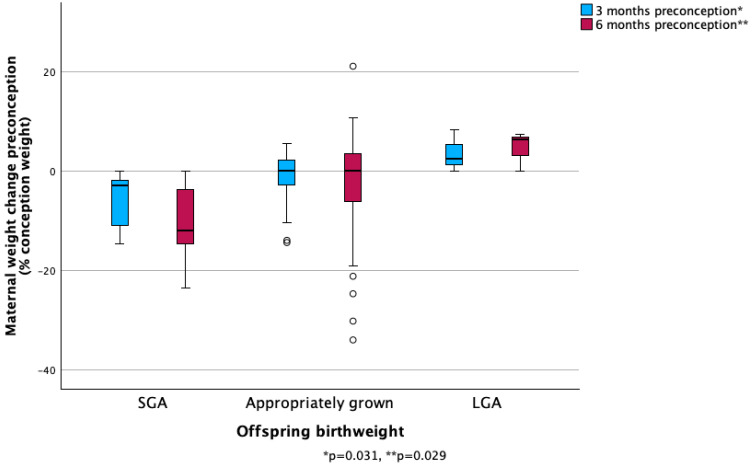
Preconception weight gain and offspring intrauterine growth.

Seven percent (5/68) of offspring were SGA and 4% (3/68) were LGA. Women who gave birth to an SGA infant had a median weight loss of −3.0 (12)% at three months and −11.9 (17)% six months preconception. In contrast, women who gave birth to LGA infants were gaining weight in the same periods, with median increases of 17.7% (IQR not available due to sample size) at three and 6.3% (IQR not available due to sample size) at six months preconception. These differences were statistically significant (*p* = 0.031 and *p* = 0.029, respectively; Figure 2). Median surgery-to-conception intervals were similar among women who birthed SGA (15 (51) months), appropriately grown (38 (50) months) and LGA neonates (27 months, IQR not available, *p* = 0.255).

### 3.2. Preterm Birth

The preterm birth rate was 7% (5/68). One participant who experienced preterm birth had a previous history of preterm birth. Surgery-to-conception interval (*p* = 0.653) and weight change at three and six months preconception were similar between women who birthed preterm and at term (*p* = 0.662 and *p* = 0.756, respectively). Gestational age at birth was not correlated with weight change at three (R = −0.12, *p* = 0.355) or six months preconception (R = −0.06, *p* = 0.618), or surgery-to-conception interval (R = −0.23, *p* = 0.059). Gestational age at birth was similar between different bariatric surgery types (*p* = 0.465). Linear regression revealed gestational hypertension as the only predictor of gestational age at birth (*F*(1, 57) = 13.914, *p* < 0.001), accounting for 18% of the variation (R^2^ = 0.443, adjusted R^2^ = 0.182). Participants with gestational hypertension gave birth significantly earlier than those without hypertension (*t =* −3.73, *p* < 0.001, β = −0.443). Participants with gestational hypertension either underwent induction of labor (50%, 3/6) or had a caesarian section prior to labor (50%, 3/6). GDM (*t =* 0.654, *p* = 0.516), smoking (*t =* 0.401, *p* = 0.690), weight change six months preconception (*t* = −0.763, *p* = 0.449) and surgery-to-conception interval (*t =* −0.367, *p* = 0.715) were excluded from the model.

## 4. Discussion

In this multicenter Australian study, we found that delaying pregnancy after bariatric surgery by at least 12 months increased the proportion of participants who were weight-stable before conception. However, weight trajectory varied between participants and only 57% of those who delayed pregnancy by 12 months were approximately weight-stable 6 months preconception. Preconception weight change, rather than interval from surgery, had a linear relationship with birth weight. Women who gave birth to SGA neonates were more likely to have lost weight in the six months preceding conception, whereas women who gave birth to LGA neonates were more likely to have gained weight during this period. Preterm birth occurred in 7% of participants but was not associated with preconception weight trajectory or bariatric surgery-to-conception interval. The findings from this small, exploratory study suggest that research examining ideal timing of pregnancy following bariatric surgery should consider the role of preconception weight trajectory. If confirmed in larger, more diverse cohorts with prospectively measured weight data, these findings may support a more individualized approach to conception timing following bariatric surgery [5,10,11].

Although rates of preterm birth and SGA infants in this study were comparable to Australian population data [29], meta-analyses suggest that the rate of SGA infants and preterm birth among bariatric surgery recipients is approximately 50% and 70% higher, respectively, compared to BMI-matched controls [1]. Whether delaying pregnancy after surgery addresses these risks is unknown. Shorter intervals (<12 or 18 months) appear associated with gestational weight gain below recommendations, whereas longer surgery-to-conception intervals are linked with weight gain above recommendations [30,31]. Our previous study clarified this relationship by determining that preconception weight changes, not interval from surgery, predicted overall gestational weight gain [21]. Despite this, two recent reviews concluded that overall pregnancy outcomes were similar across surgery-to-conception intervals of 12, 18, 24 and 48 months [30,32]. This conclusion, however, was based primarily on small, retrospective studies with methodological limitations. Notably, two large well-designed studies have reported lower birth weight and gestational age in women who conceived within 12 months [33] and within 24 months of surgery [34]. Our findings suggest that these outcomes may be influenced by weight changes prior to conception.

The observation that preconception weight status influences birth weight is biologically plausible. Birth weight generally increases with maternal BMI, which is thought to reflect greater insulin resistance, increasing glucose availability, and larger placental sizes in women with higher BMI [35]. Weight loss exceeding 1 kg/m^2^ between pregnancies in women without bariatric surgery has been associated with increased SGA risk [35]. Mechanisms for this may include improved glycemic control, limiting glucose availability to the fetus [35]. Weight loss may also reflect lower dietary intake, potentially leading to micronutrient deficiency, thereby decreasing availability of nutrients needed for fetal growth [36]. In our study, participants reported a median loss of 15 kg/m^2^ during the 31 months between surgery and conception. A 2022 scoping review of six studies evaluating the relationship between total post-operative weight loss and birth weight reported conflicting findings [32]. Studies reported greater post-operative weight loss either increased (three studies), reduced (one study) or did not impact birth weight (two studies) [32]. These varying results may be explained by weight regain, which occurs in 20 to 40% of cases, and can begin from 6 months up to 3 years after bariatric surgery [37,38]. In our study, we observed significant variation in preconception weight trajectories. Just over half (57%) of women who conceived after 12 post-operative months were weight-stable, suggesting that a significant proportion (~43%) were not in energy balance, either losing or regaining weight. This differed from Hazart et al. [12] who reported that 84% of participants were weight-stable if they conceived ≥18 months post-operatively. However, the methods used by Hazart et al. [12] to define weight stability are unclear, which may have contributed to this inconsistency. There is no doubt that there are large differences in weight loss trajectories among women post-bariatric surgery, and this study suggests that weight changes leading up to conception are of clinical importance [12,37,38].

International consensus guidelines recommend multidisciplinary care, additional ultrasound scans, micronutrient supplementation and monitoring for deficiencies during pregnancy for all women with a history of bariatric surgery alongside routine antenatal care [5]. Although dietetic care is recommended for this cohort of women, evidence to guide nutrition interventions is scarce [17]. The findings of our study suggest that considering weight trajectory preconception could inform more tailored dietary interventions. Women who lose weight in the months preceding conception may be at an increased risk of delivering an SGA infant. In contrast, those gaining weight at conception may be more likely to deliver a LGA infant. Medial nutrition therapy approaches to address these risks vary. Women at risk of SGA infants may benefit from increased monitoring for micronutrient deficiencies and signs of maternal malnutrition [39]. Interventions that support adequate gestational weight gain, micronutrient supplementation, appropriate macronutrient intake and alignment with some dietary patterns (such as the Mediterranean diet) can be effective at reducing the risk of SGA [39]. Conversely, reducing the risk of LGA offspring may require increased surveillance for GDM or maternal hyperglycemia, supporting physical activity, and promoting dietary patterns high in fiber and low in saturated fat [36]. Although these interventions have not been evaluated in women with a history of bariatric surgery, incorporating preconception weight trajectory into future studies may provide a more comprehensive understanding of their effectiveness and support more cost-effective, woman-centered care.

This study is strengthened by its prospective study design and recruitment of a pragmatic sample of women receiving routine care in four Australian hospitals. As with any observational study, however, there are several limitations to consider. Firstly, the small sample size reduced statistical power and the precision of regression analysis. It also prevented adjustment for potentially important mediators of perinatal outcomes, including gestational weight gain, dietary intake, and micronutrient status, and meant analysis could not be stratified by bariatric procedure. Consequently, the findings should be interpreted cautiously and require confirmation in larger studies. Secondly, preconception weight was obtained by retrospective self-report, introducing the potential for recall bias. Underreporting of self-reported weight is common, but is typically modest (<1.5 kg), and studies have demonstrated similar associations between self-reported and measured maternal weight and perinatal outcomes [40,41,42,43]. As objective preconception weight measurements were unavailable, self-reported weight represented the only feasible approach for assessing preconception weight trajectory. Self-reported preconception weight has also not been specifically validated among women following bariatric surgery. Consequently, some measurement error in the assessment of preconception weight is likely. Finally, the external validity of these findings may be limited. Meta-analyses suggest that risks of SGA and preterm birth are higher following gastric bypass procedures compared to restrictive surgeries; however, this study included primarily sleeve gastrectomy recipients [1]. Participants were recruited from Australian maternity hospitals where multidisciplinary care was routinely available, which has been associated with improved dietary behavior and health outcomes [20]. These factors may have contributed to the more favorable outcomes observed in this cohort, particularly for the incidence of SGA neonates, compared to previously published studies [31,44]. The findings may therefore not be generalizable to women receiving care in different healthcare systems or in settings without access to specialist bariatric services.

## 5. Conclusions

This study suggests that maternal weight trajectory in the months preceding conception may influence offspring growth, independent of the time elapsed since bariatric surgery. Greater preconception weight loss was associated with lower birth weight, whereas weight gain appeared to increase offspring birthweight. Guidelines [10,11] that recommend delaying pregnancy for 12–24 months post-surgery may overlook important individual variations in nutritional status and weight trajectory. These findings should be interpreted cautiously given the study’s small sample size and methodological limitations; however, they suggest that preconception weight trajectory following bariatric surgery may be an important factor to consider in future research. If confirmed in larger, more representative studies, preconception weight trajectory could help inform more individualized preconception counseling and dietetic care during pregnancy. Ultimately, this knowledge may contribute to improved pregnancy outcomes, more efficient use of healthcare resources, and care that better aligns with consumer preferences [17]. Future research should validate methods to measure preconception weight changes and explore the relationship between weight trajectory, dietary intake, and metabolic health in larger Australian cohorts to inform the development of evidence-based guidelines for pregnancy after bariatric surgery.

## Figures and Tables

**Table 1:** Participant demographics.

Characteristic	Percent (n=)
Maternal age at conception (years)	32.1 ± 4.8 ^1^
Parity	1 (2) ^2^
Nulliparous, % (n=)	37% (25/68)
Ethnicity, % (n=)	
Caucasian	78% (52/68)
Aboriginal or Torres Strait Islander	10% (7/68)
Pacific Islander	3% (2/68)
Other	9% (6/68)
Bariatric surgery-to-conception interval (months)	31 (50) ^2^
Bariatric surgery type, % (n=)	
Gastric band	2% (1/68)
Sleeve gastrectomy	68% (46/68)
Gastric bypass	31% (21/68)
Revisional surgery % (n=)	7% (5/68)
Marital status, % (n=)	
Married or de facto	66% (44/68)
Never married	27% (18/68)
Divorced or separated	8% (5/68)
Education attainment, % (n=)	
Postgraduate or undergraduate qualification	24% (16/68)
Trade, technical certificate or diploma	39% (26/68)
Completed schooling	28% (19/68)
Did not complete schooling	9% (6/68)
Smoking reported during pregnancy, % (n=)	16% (10/64) ^3^
Alcohol use reported during pregnancy, % (n=)	13% (8/63) ^4^
Assisted conception, % (n=)	5% (3/65) ^5^
History of preterm birth, % (n=)	5% (3/59) ^6^

^1^ Mean ± SD, ^2^ Median (IQR), ^3^ 4/68 missing, ^4^ 5/68 missing, ^5^ 3/68 missing, ^6^ 9/68 missing.

**Table 2:** Maternal anthropometry during pregnancy after bariatric surgery.

	All Participants (N = 68)		<12 Months Post-Surgery (n = 12)	≥12 Months Post-Surgery (n = 55)	
Variable	Median (IQR)	Range	Median (IQR)	Median (IQR)	*p*-Value
Pre-bariatric surgery BMI (kg/m^2^)	45.0 (13)	33–73	43.4 (12)	45.1 (13)	*p* = 0.335
Weight change 3 months preconception (%) ^1^ Weight stable ±5%, % (n=)	0 (5) 79% (46/59)	−15–8	−10.1 (6) 18% (2/12)	0.0 (4) 93% (43/55)	***p < 0.001***
Weight change 6 months preconception (%) ^2^ Weight stable ±5%, % (n=)	0 (11) 48% (30/64)	−34–21	−19.1 (15) 9% (1/12)	0.0 (4) 57% (29/55)	***p < 0.001***
BMI at conception (kg/m^2^)	30.5 (7.4)	18.4–50.4	32.0 (7.5)	30.1 (7.9)	*p* = 0.258
BMI category, % (n=) ^2^					
BMI <18.5	2% (1/64)		0% (0/12)	2% (1/55)	
BMI 18.5–24.9	14% (9/64)		0% (0/12)	17% (9/55)	
BMI 25–29.9	28% (18/64)		27% (3/12)	28% (15/55)	
BMI >30	58% (37/64)		73% (8/12)	53% (28/55)	
Gestational weight gain (kg) ^3,4^	7.4 ± 7.3	−12–22	−0.2 ± 3.7	8.4 ± 7.1	***p = 0.006***
Alignment with gestational weight gain recommendations ^3^, % (n=)					
Below recommendations	33% (16/49)		83% (5/6)	26% (11/42)	
Within recommendations	30% (14/49)		17% (1/6)	31% (13/42)	
Above recommendations	38% (18/49)		0% (0/6)	43% (18/42)	

^1^ 9/68 unsure, ^2^ 4/68 unsure, ^3^ 9/68 unsure or gave birth before responding, ^4^ Mean ± SD. bold/italics required to highlight significant *p*-values.

**Table 3:** Complications of pregnancy and offspring birth weight.

Outcome	Percent (n=)
GDM ^1^	15% (10/66)
Gestational hypertension Pre-eclampsia	9% (6/68) 3% (2/68)
Onset of labor	
Spontaneous	37% (25/68)
Induced	38% (26/68)
No labor	25% (17/68)
Mode of delivery ^2^	
Vaginal birth	49% (33/67)
Vaginal instrument-assisted birth	9% (6/67)
Cesarean section	42% (28/67)
Gestational age at birth (weeks, median (IQR)) <37 weeks’ gestation	39 (2.1) 7% (5/68)
Birth weight (grams, median (IQR))	3368 (514)
SGA (5/68)	2510 (310)
LGA (3/68)	3945 ^3^
Offspring sex (% female)	47% (32/68)

^1^ 2/68 missing, ^2^ 1/68 missing, ^3^ insufficient sample size to compute IQR.

## Data Availability

The data presented in this study are available on request only from the corresponding author due to privacy and ethical reasons.

## Supplementary Materials

The following supporting information can be downloaded at: https://www.mdpi.com/article/10.3390/jcm15155851/s1, Table S1: Indications for caesarian section.

## Abbreviations

The following abbreviations are used in this manuscript:

BMI	Body Mass Index
CI	Confidence Interval
GDM	Gestational Diabetes Mellitus
LGA	Large-for-Gestational-Age
OR	Odds Ratio
SGA	Small-for-Gestational-Age
