# Supplementary material for: Preconception Weight Change and Pregnancy Outcomes After Bariatric Surgery
Source: J Clin Med. 2026 Jul 27;15(15):5851. doi: 10.3390/jcm15155851 (PMC13467303; doi:10.3390/jcm15155851)
Supplement: Supplementary file 1 [file jcm-15-05851-s001.zip › jcm-4383960-supplementary.pdf]

Supplementary material

Table S1. Indications for caesarian section

Indication	Percent (n=)
Elective	43% (12/28)
Failure to progress	14% (4/28)
Breech presentation	7% (2/28)
Fetal distress	14% (4/28)
Repeat caesarian section	11% (3/28)
Intrauterine growth restriction	4% (1/28)
Umbilical cord prolapse	4% (1/28)
Not reported	4% (1/28)
